# Probability of Target Attainment of Tobramycin Treatment in Acute and Chronic *Pseudomonas aeruginosa* Lung Infection Based on Preclinical Population Pharmacokinetic Modeling

**DOI:** 10.3390/pharmaceutics14061237

**Published:** 2022-06-11

**Authors:** Bruna Bernar Dias, Fernando Carreño, Victória Etges Helfer, Priscila Martini Bernardi Garzella, Daiane Maria Fonseca de Lima, Fabiano Barreto, Bibiana Verlindo de Araújo, Teresa Dalla Costa

**Affiliations:** 1 Pharmaceutical Sciences Graduate Program, Federal University of Rio Grande do Sul–UFRGS, Porto Alegre 90610-000, Brazil; b.bernardias@gmail.com (B.B.D.); vehelfer@gmail.com (V.E.H.); priscila.bernardi@hotmail.com (P.M.B.G.); daianefonsecalima@gmail.com (D.M.F.d.L.); bibiana.araujo@ufrgs.br (B.V.d.A.); 2 Eshelman School of Pharmacy, University of North Carolina, Chapel Hill, NC 27599, USA; carrenof@email.unc.edu; 3 Federal Laboratory of Animal and Plant Health and Inspection–LFDA/RS, Porto Alegre 90610-000, Brazil; fabiano.barreto@agricultura.gov.br

**Keywords:** tobramycin, popPK model, *Pseudomonas aeruginosa* infection model, biofilm, microdialysis, PTA

## Abstract

Biofilms and infectious process may alter free antimicrobial concentrations at the site of infection. Tobramycin (TOB), an aminoglycoside used to treat lung infections caused by *Pseudomonas aeruginosa*, binds to alginate present in biofilm extracellular matrix increasing its minimum inhibitory concentration (MIC). This work aimed to investigate the impact of biofilm-forming *P. aeruginosa* infection on TOB lung and epithelial lining fluid (ELF) penetration, using microdialysis, and to develop a population pharmacokinetic (popPK) model to evaluate the probability of therapeutic target attainment of current dosing regimens employed in fibrocystic and non-fibrocystic patients. The popPK model developed has three compartments including the lung. The ELF concentrations were described by a penetration factor derived from the lung compartment. Infection was a covariate in lung volume (V3) and only chronic infection was a covariate in central volume (V1) and total clearance (CL). Simulations of the recommended treatments for acute and chronic infection achieved >90% probability of target attainment (PTA) in the lung with 4.5 mg/kg q24h and 11 mg/kg q24h, respectively, for the most prevalent *P. aeruginosa* MIC (0.5 mg/mL). The popPK model was successfully applied to evaluate the PTA of current TOB dosing regimens used in the clinic, indicating the need to investigate alternative posology.

## 1. Introduction

Lung infections usually start with bacterial contamination through the upper respiratory tract until reaching the lungs. Biofilm presence in acute and chronic lung infections caused by *Pseudomonas aeruginosa* confer tolerance to antimicrobial bacterial penetration, leading to an increase of up to one thousand times in the minimum inhibitory concentration (MIC) against susceptible bacteria growing in biofilms when compared to the planktonic form of growth [1,2,3]. In addition to biofilms, acute and chronic infectious processes cause several physiological changes in the host site of infection such as increased tissue temperature and protein concentration, decreased pH, and leukocyte migration, which can alter the free antimicrobial concentrations at the biophase [4].

Owing to the route of contamination, free concentrations of antimicrobials in the pulmonary epithelial lining fluid (ELF), in addition to free concentrations in the lung tissue, are relevant to the treatment of acute and chronic lung infections. The penetration of drugs from the blood into the pulmonary interstitial space and deeper into the bronchial space can be hampered by the different barriers that separate the pulmonary compartments. The vascular endothelium tends to be more permeable to hydrophilic drugs, such as tobramycin (TOB), than the alveolar membrane, generating different concentrations along the path taken by the drug to reach the bronchus from the blood stream, following intravenous dosing [5,6,7].

Tobramycin is an aminoglycoside used in cystic fibrosis (CF) patients as prophylaxis to *P. aeruginosa* chronic colonization and acute exacerbation [8,9]. However, it has been shown in vitro that TOB binds to alginate present in biofilm extracellular matrix increasing its MIC 900 times [10]. Tobramycin dosing regimens used to treat *P. aeruginosa* lung infections are a 30 min intravenous infusion of 1–3 mg/kg q8h for non-CF patients and 10 mg/kg, or most recently recommended 11 mg/kg [11], q24h for CF patients. Once daily (q24h) dosing is preferred for CF patients, and it is more efficacious than the total dose administered three times a day recommended for non-CF patients. The therapeutic targets for total plasma concentrations are: through levels ≤ 1 mg/L (q24h) and ≤2 mg/L (q8h) and peak levels in the range 20–40 mg/L (q24h) and 5–20 mg/L (q8h) [12]. Even though drug monitoring is applied to maintain plasma levels within the target range, currently it does not take into consideration that TOB binds to the biofilm’s alginate in the target tissue, increasing tolerance to the drug and, consequently, leading to therapeutic failure [13,14].

Biofilm in acute and chronic *P. aeruginosa* infections can present different extracellular matrix components. The acute infection bacteria produce an extracellular matrix with full chains of lipopolysaccharide (LPS) and extracellular DNA (e-DNA). Alginate is overexpressed in *P. aeruginosa* strains that mostly compose the chronic infections, resulting in their mucoid phenotype [15]. Cystic fibrosis patients present a high amount of mucus-containing alginate in the airways, which benefits the maintenance of mucoid *P. aeruginosa* survival and the chronicity of the infection, making it difficult for bacterial eradication [13].

Although TOB plasma pharmacokinetics is well characterized, free TOB concentrations at the biophases—lung interstitial space and epithelial lining fluid—in healthy and infected individuals have not been reported. Therefore, this work aimed to investigate the impact of biofilm-forming *P. aeruginosa* infection on TOB lung and ELF penetration, using microdialysis, and to develop a population pharmacokinetic (popPK) model to evaluate the probability of therapeutic target attainment (PTA) of the current dosing regimens employed in CF and non-CF patients.

## 2. Materials and Methods

### 2.1. Chemicals and Reagents

Tobramycin was obtained from Ontario Chemicals (Canada) (>98.0%). Apramycin (internal standard-IS) (>95.0%) was purchased from Dr. Ehrenstorfer (Augsburg, Germany). Acetonitrile HPLC grade was obtained from JT Baker (Phillipsburg, NJ, USA). Heptafluorobutyric acid (HFBA), trichloroacetic acid (TCA), urethane (ethyl carbamate), and alginic acid sodium salt from brown algae were obtained from Sigma-Aldrich (São Paulo, Brazil). Tris(hydroxymethyl)aminomethane was purchase from Neon (Suzano, Brazil). Mueller–Hinton agar and broth were obtained from Kasvi (Roseto Degli Abruzzi, Itália). Heparin (5000 IU/mL) was purchased from Cristália Produtos Químicos Farmacêuticos (São Paulo, Brazil). Ketamine and xilazine were purchase from Sespo Indústria e Comércio Ltda. (Paulinia, Brazil). Sodium chloride, calcium chloride, potassium chloride, and all other chemicals and reagents were of analytical grade and purchased from commercial sources. Ringer’s solution used in microdialysis consisted of 147 mM NaCl, 1.3 mM CaCl2, and 4 mM KCl.

### 2.2. Animals

This study was approved by the Ethics Committee in Animal Use from the Federal University of Rio Grande do Sul (UFRGS/CEUA #32345) and was conducted in compliance with the principles of laboratory animal care from the National Council for Animal Experimentation (CONCEA/Brazil). Male Wistar rats (200–250 g) were obtained from the Center for Laboratory Animals Reproduction and Experimentation (CREAL) of Federal University of Rio Grande do Sul (Porto Alegre, Brazil). All animals were kept in a controlled environment (22 ± 2 °C, 65% humidity, in a 12 h light/dark cycle) with free access to standard rodent chow and filtered water.

### 2.3. Preclinical Model of Acute and Chronic P. aeruginosa Lung Infection

A bacterial suspension containing 10^9^ CFU/mL of *P. aeruginosa* PA14 in sterile saline solution (0.9%) was prepared to establish the acute infection. Briefly, 30 μL of frozen bacteria in skim-milk was incubated in 30 mL of Mueller–Hinton broth for 18 h at 37 ± 1 °C. A bacterial loop was seeded in an inclined tube of Mueller–Hinton agar and incubated for 18 h at 37 ± 1 °C. To prepare the bacterial suspension on the day of inoculation, 5 mL of sterile saline solution (0.9%) was added to the tube and the absorbance at 600 nm measured in a spectrometer and adjusted for 0.90 ± 0.02. The bacterial suspension was diluted and plated in Mueller–Hinton agar plates to confirm the CFU/mL. Chronic infection was developed to mimic the chronic infection observed in CF patients following previously reported protocols [16,17,18,19]. In order to produce a chronic infection, bacteria was impregnated in alginate beads according to Torres et al. (2017) [20] based on Johansen & Høiby (1999) [21]. Briefly, 1 mL from a bacterial suspension, prepared as previously described, containing 10^9^ CFU/mL of *P. aeruginosa* ATCC 27,853 was mixed with 9 mL of sterile alginate solution (1 mg/mL) and sprayed into a constant-stirring 0.1 M CaCl_2_ solution in tris-HCL buffer (0.1 M, pH 7.0). After 1 h, the alginate beads were centrifuged at 180× *g* for 10 min and washed twice with sterile saline solution. Blank beads, without bacteria, were also prepared using the same procedure.

Male Wistar rats were intraperitoneally (i.p.) anesthetized with ketamine–xylazine (100 and 10 mg/kg, respectively), and inoculated intratracheally with 100 or 50 µL of bacteria suspension or beads containing bacteria for developing the acute or chronic infection, respectively. Blank beads were also inoculated in the control groups (50 μL) in the same manner. For acute infection group, the pharmacokinetic experiments were conducted 7 days after inoculation, whereas for chronic infection group, 14 days later. After pharmacokinetic experiments, animals were euthanized by anesthetic (ketamine–xylazine) overdose; their lungs were surgically removed, homogenized in sterile saline, and plated in Muller–Hilton agar plates to confirm the presence of infection by identification of *P. aeruginosa* colonies after 24 h incubation at 37 ± 1 °C.

### 2.4. Pharmacokinetic Experiments

#### 2.4.1. Tobramycin Plasma Concentration–Time Profiles

Tobramycin plasma concentrations were determined in healthy (n = 6), blank-bead (n = 4), and acutely (n = 5) and chronically (n = 6) infected animal groups following 10 mg/kg i.v. bolus dosing through the femoral vein. Blood samples (~200 μL) were collected from anesthetized (urethane, 1.25 g/kg i.p.) Wistar rats, by a surgically implanted cannula in the carotid artery, into heparinized microtubes at pre-determined times after dosing (0.08, 0.25, 0.5, 0.75, 1, 2, 4, 6, 8, and 12 h) and immediately centrifuged (20,000× *g* at 4 ± 1 °C, 10 min). Plasma was stored at −80 ± 2 °C until analysis.

For TOB quantification, plasma proteins were precipitated using 15% trichloroacetic acid (TCA) in acetonitrile-water (50:50, *v*/*v*), centrifuged, and the supernatant analyzed to quantify total TOB concentrations using a previously validated HPLC-MS/MS method [22]. Microdialysate samples were analyzed without prior treatment. A brief description of the analytical method and validation is presented in the Supplementary Materials.

#### 2.4.2. Free Lung and Free ELF Pharmacokinetic Experiments

Microdialysis was used to determine TOB free concentrations in lung and ELF of healthy (n = 6 and 12, respectively), blank-bead (n = 5 and 5, respectively), and acutely (n = 5 and 6, respectively) and chronically (n = 6 and 3, respectively) infected groups.

For lung microdialysis, anesthetized (urethane, 1.25 g/kg i.p.) animals were placed in a supine position, intubated by tracheotomy, and placed under artificial ventilation using a rodent respirator (2.5 of air volume and 64–68 min^−1^ frequency, Harvard Apparatus, Holliston, MA, USA). The right lung was exposed and the CMA/20 microdialysis probe (4 mm, cutoff 20 kDa, CMA^®^, Gothenburg, Sweden) was carefully inserted into the intermediate lobe through a gentle incision made with a needle. For conducting ELF microdialysis, a custom-made CMA/20 probe (2 mm, cutoff 20 kDa, CMA^®^, Gothenburg, Sweden) was inserted through the rat trachea by tracheotomy. During the ELF microdialysis, the anesthetized animals (urethane, 1.25 g/kg i.p.) did not receive breathing support.

Microdialysis probes were connected to a PHD 2000 syringe pump (Harvard Apparatus, Holliston, MA, USA) to perfuse Ringer solution at a flow rate of 1.0 µL/min. After 1 h of probe stabilization, followed by in vivo relative-recovery determination (see Section 2.4.3), animals received TOB 10 mg/kg i.v. bolus dose through the femoral vein, and microdialysate samples were collected every 30 min for up to 12 h. Considering the void probe and tubing volumes, a delay of 7 and 17 min for lung and ELF microdialysis, respectively, was observed before starting microdialysate collection. Samples were stored at −80 ± 2 °C until analysis, performed by a previously validated HPLC-MS/MS method [22].

#### 2.4.3. In Vivo Microdialysis Probe Calibration

In vitro relative recoveries previously reported indicated that TOB does not bind to microdialysis probes and tubing, and that the drug recovery is concentration-independent [22]. For correct measurement of free TOB concentrations in the microdialysis experiments, probes were previously calibrated in vivo by retrodialysis. After the probe was inserted into the rats’ lung or trachea, it was perfused with a 10 µg/mL TOB in Ringer’s solution at a perfusion flow rate of 1 µL/min for 1 h for stabilization, before three microdialysate samples were collected at 30 min intervals. Tobramycin concentrations in microdialysate samples and perfusate solutions were quantified and the probes’ in vivo relative recovery (RR) was individually calculated using specific equations [22]. The real free TOB concentrations for each tissue of each animal used to build the model were corrected by the value of the respective probe´s relative recovery.

### 2.5. Population Pharmacokinetic (popPK) Model Building

Data analysis was performed by the population approach using NONMEM^®^ version 7.4 (Icon Development Solutions, Ellicott City, MD, USA) and PsN version 4.9.0 software. (Perl-speaks-NONMEM, Uppsala, Sweden) All estimations applied the first-order conditional estimation method with interaction (FOCE-I). Model management was done in PIRANA^®^ version 3.0.0 (Pirana Software and Consulting, Certara, Princeton, NJ, USA). Ggplot2 and xpose4 libraries for R program, version 4.1.1 and RStudio, version 1.4.1717 (The R Foundation for Statistical Computing, Vienna, Austria) were used for graphical analysis.

Data below the quantification limit (BQL) and missing data were excluded from the analysis since they represented less than 5% of the total observations (52 observations, 0.25%). Free plasma concentrations were calculated using the plasma protein-binding of 11%, determined experimentally [22]. Microdialysate data were described by the integral over-each collection interval. Therefore, no assumptions regarding collection times were made. An exploratory analysis of TOB time–concentration curves was conducted to detect outliers and to identify potential pharmacokinetic models and covariates (Supplementary Materials Figure S1).

Structural model-building was performed sequentially. First the observed TOB plasma concentrations obtained from healthy rats were fitted to a two-compartment model. The model was then expanded to include the free lung and free ELF concentrations. In the sequence, acute, chronic, and blank-bead, data were included in the model. The model was parameterized by differential equations using an ADVAN 13 TRANS1 subroutine. Thus, inter-individual variability (IIV) was modeled exponentially, and residual variability was described separately for plasma and microdialysate data with a log-additive error model.

Body weight, infection condition (acute or chronic), and inoculation of blank bead were analyzed as covariates. Since body weight did not show correlation in the exploratory analysis performed in the basic model, only the different infection conditions and inoculation of blank-bead were evaluated as a covariate in the model, using the stepwise covariate method (SCM) (Supplementary Materials Figures S2 and S3). Model selection was guided by: (i) significant changes in the value of the objective function (OFV), considering the evaluation of two models, a decrease of 3.84 or 6.64 points in OFV (*p* < 0.05 or *p* < 0.01) was considered as statistically significant; (ii) visual exploration of goodness-of-fit (GOF) plots; and (iii) precision of model parameters, reflected as the relative standard error (RSE) computed as the ratio between the standard error and the parameter estimate.

Evaluation of the selected popPK model was performed according to GOF plots, conditional number, and %RSE and through prediction-corrected visual predictive checks (pcVPC). For each experimental group—healthy, acute, chronic, and blank-bead—of plasma, lung, and ELF, one thousand simulated profiles were generated and the 10th, 50th, and 90th percentiles were calculated and displayed graphically together with the experimental data. To check model stability, a non-parametric bootstrap analysis was performed for the final model (n = 1000), obtaining the medians and confidence intervals (CIs) of the 5th and 95th quartiles.

### 2.6. Probability of Target Attainment

To investigate if current TOB dosing regimens to treat *P. aeruginosa* pneumonia are effective, the popPK model developed was used to simulate free lung and ELF concentrations and to determine the PTA of this treatment in humans. Initially, the doses used to treat lung infections in patients were allometrically scaled from humans to rats [23]. The equations used (Equations (S1)–(S2)) and the resulted doses (Table S1) for simulations are presented in the Supplementary Materials. Then, 1000 simulation of free lung and ELF TOB concentrations were performed using the popPK model and the concentrations obtained were compared with clinical data previously reported by Boselli et al. (2007) [24], who evaluated free plasma and free ELF concentrations in cystic fibrosis patients after a 10 mg/kg i.v. dosing. In sequence, the pharmacokinetic/pharmacodynamic (PK/PD) index free peak concentrations over MIC (*f*C_max_/MIC) higher than 10 [25] were used to assess the PTA in 1000 simulations for different therapeutic regimes using the EUCAST [26] database for *P. aeruginosa* MIC (from 0.016 to 512 mg/L). A PTA > 90% was assumed as adequate clinical outcome.

The regimes tested in the simulations were based on those reported in the literature for treating acute and chronic infection by *P. aeruginosa*: 30 min i.v. infusion of 1 mg/kg every 8 h, 3 mg/kg every 24 h, and 11 mg/kg every 24 h for CF patients [11,25,27].

## 3. Results

Microdialysis relative recoveries determined by retrodialysis were 27.7% ± 7.7 for lung probes and 26.6% ± 9.8 for ELF probes, independent of the animal’s tissue condition (healthy or infected). Despite the length difference between the probes (4 mm for lung and 2 mm for ELF, respectively), which would anticipate a smaller recovery for ELF probes, the tortuosity and smaller volume in the lung tissue could be responsible for the similarity observed in the relative recoveries.

A total of 1231 observations from a total of 71 rats was used for building the population pharmacokinetic model. A summary table of individuals/groups and respective number of observations is provided in Supplementary Materials (Table S2). Alginate beads impregnated with bacteria were used to avoid bacterial clearance from the lungs in chronic infection groups. Knowing that TOB binds to alginate in vitro, reducing its pharmacological availability [13,14], blank-bead groups were used as control to investigate the influence of alginate on TOB tissue distribution. Raw plasma and tissue concentration–time profiles for the different groups can be observed in Figure S1.

**Figure 1:**
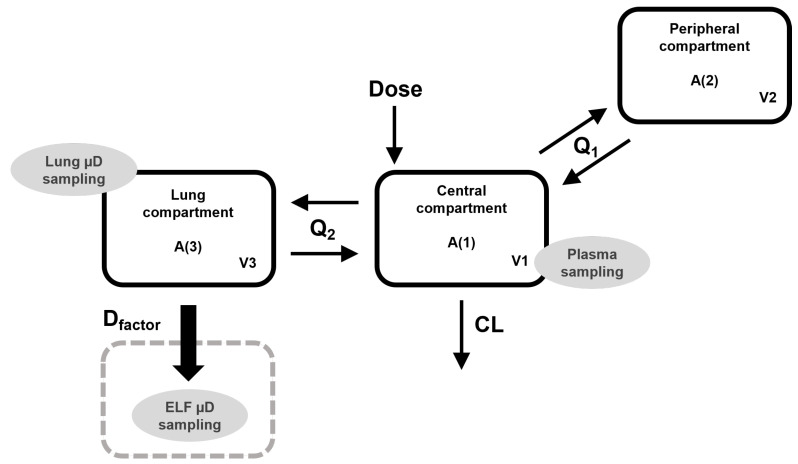
Schematic representation of the structure of the final TOB popPK model.

The tobramycin base structural model, represented in Figure 1, is a three-compartmental model with linear elimination from the central compartment, parameterized as distribution volumes (V1, V2, and V3), intercompartmental clearances (Q1 and Q2), and total clearance from the central compartment (CL). The lung is represented as the third compartment, where microdialysate samples were collected. Epithelial lining fluid microdialysate concentrations were incorporated into the model as a fraction of the lung compartment, described by a distribution factor (D_factor_). Other structural models tested were the inclusion of lung as the peripheral compartment or lung and ELF as the third and fourth compartments, respectively. The inclusion of lung as a third compartment and the description of ELF concentrations as a fraction of the lung provided the best fit for the model. Equations used to estimate free concentrations at the different compartments are described below (Equations (1)–(3)):

(1) $$\frac{dA_{\mathrm{central}}}{\mathrm{dt}}=-\left(\frac{Q_{1}}{V_{1}}+\frac{Q_{2}}{V_{1}}+\frac{\mathrm{CL}}{V_{1}}\right)\cdot A_{1}+\frac{Q_{1}}{V_{2}}\cdot A_{2}+\frac{Q_{2}}{V_{3}}\cdot A_{3}$$

(2) $$\frac{dA_{\mathrm{peripheral}}}{\mathrm{dt}}=-\frac{Q_{1}}{V_{2}}\cdot A_{2}+\frac{Q_{1}}{V_{1}}\cdot A_{1}$$

(3) $$\frac{dA_{\mathrm{lung}}}{\mathrm{dt}}=-\frac{Q_{2}}{V_{3}}\cdot A_{3}+\frac{Q_{2}}{V_{1}}\cdot A_{1}$$

where $A_{1}$, $A_{2}$, and $A_{3}$ are plasma, lung, and ELF unbound amounts of TOB, respectively; $Q_{1}$ is the intercompartmental clearance between central and peripheral compartment and $Q_{2}$ the intercompartmental clearance between the central and the lung compartment; CL is the total elimination clearance from central compartment. Free lung concentrations were calculated as $A_{3}/V_{3}$ and the free ELF concentrations as $\left(A_{3}/V_{3}\right)\cdot D_{factor}$. To compare observed plasma concentrations with the free concentrations estimated by the model, the estimates were divided by 0.89, the unbound fraction of the drug [22].

Interindividual variability (IIV) was included in CL, V1, V3, and D_factor_. The SCM analysis demonstrated that the inclusion of chronic infected condition as a covariate in CL and V1 significantly increased the model fit compared to the base model. The parameters CL and V1 were estimated separately for the chronic infected group. The estimated CL and V1 were higher in animals with chronic infection (CL_chronic_ = 0.085 L/h), compared to the healthy, acutely infected, and blank-bead groups (CL = 0.047 L/h). Acute, chronic, and blank-bead were included as covariates in V3, which was significantly increased compared to the healthy condition (0.130 L and 0.083 L, respectively). Table 1 shows the parameters estimated by the model, with a relative standard error (%RSE) no greater than 20%, and the 95th confidence intervals from the bootstrap analysis, that had 96% successful runs confirming the model stability.

**Figure 2:**
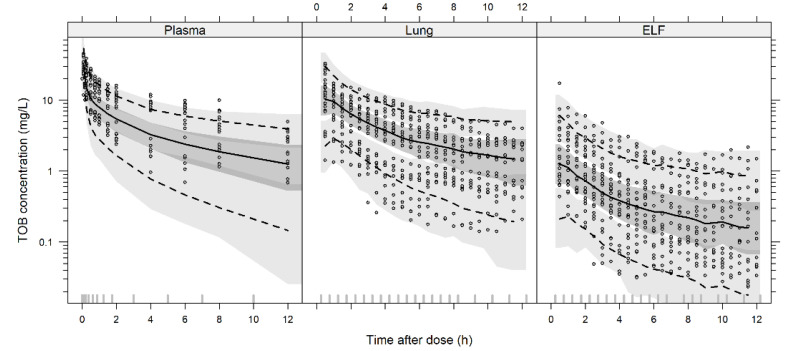
Predicted-corrected visual predictive check of the final popPK model stratified by plasma, lung, and ELF. VPCs are based on 1000 simulations and show a comparison of the observations (dots) with the 10th, 50th, and 90th percentiles of the 1000 simulated profiles (dashed lines and shadow areas).

The pcVPC (Figure 2) stratified by plasma, lung, and ELF for the final popPK model demonstrated that all predictions were consistent with the observed data presenting a good agreement between observed result and the population prediction. Goodness-of-fit plots for the final model demonstrated a good fit for the model, as well as the observed result vs. the population and individual predictions (Supplementary Materials Figures S4 and S5).

To confirm that the popPK model developed can be used to evaluate the efficacy of TOB treatments in humans, we compared concentrations simulated using the popPK model with free plasma and free ELF concentrations reported in CF patients after a 10 mg/kg dose [25]. Boselli et al. (2007) found that the TOB ELF/serum penetration ratio was around 20%, with a free C_max_ in plasma ranging from 17.2 to 39.2 mg/L, and in ELF from 1.7 to 4.4 mg/L. Model simulation of the allometrically scaled human dose to rats showed similar TOB free concentrations in plasma and ELF, with a penetration ratio of 13%. The confidence interval of the free concentration simulated in plasma, for the same time-points, ranged from 14.5 to 20.5 mg/L and in ELF from 1.48 to 4.16 mg/L. A graphical comparison of the literature data and the simulated profile, presented in the Supplementary Materials (Figure S6), shows that the observations from humans are inside the prediction interval of 32nd and 68th for the simulated model. This comparative analysis proves that the model developed with preclinical data, even though it may not be directly applied to clinical practice, can be used to infer free TOB concentrations expected in humans using allometrically scaled doses.

**Figure 3:**
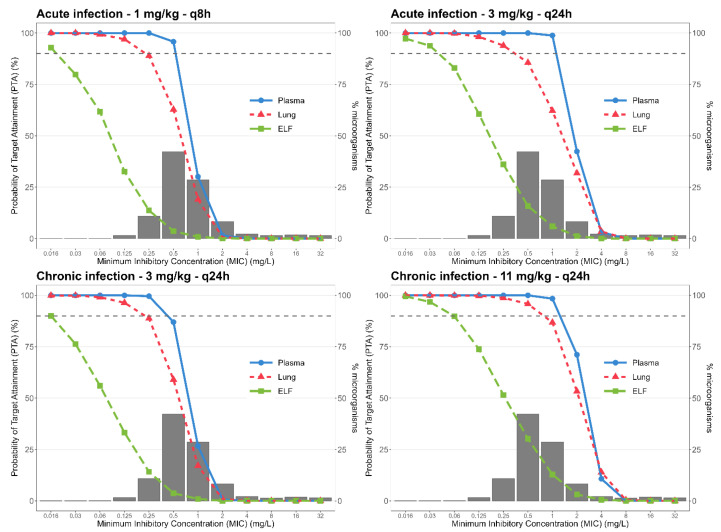
Probability of target attainment for a PK/PD index of *f*C_max_/MIC > 10 for *P. aeruginosa* for different TOB dosing regimens considering free plasma, free lung, and free ELF concentrations.

To assess the PTA of different dosing regimens used in clinical practice, simulations of 1000 profiles using the final popPK model were conducted to evaluate different allometrically scaled TOB dose regimens (Table S1)**.** Figure 3 presents the PTA of these regimens against the MIC distribution for *P. aeruginosa* for free plasma, free lung, and free ELF concentrations considering acute and chronic infections. The target PK/PD index used was TOB *f*C_max_/MIC greater that 10 [25] with a probability >90%.

For acute infection, the recommended TOB regimen of 1 mg/kg q8h achieved 90% PTA for the most prevalent *P. aeruginosa* MIC (0.5 mg/L) only for free plasma concentrations, while the PTA for free lung concentrations was 62.8%. Once-a-day 3 mg/kg dosing resulted in 100% PTA for free plasma and 85.6% PTA for free lung concentrations considering the most prevalent MIC. Both regimes showed a very low PTA for TOB in ELF, 3.6% and 15.8%, for 1 mg/kg q8h and 3 mg/kg q24h, respectively.

The PTA for chronic infection using TOB 3 mg/kg q24h was 87.0% for free plasma, 59% for free lung, and 3.7% for free ELF concentrations. The regimen of 11 mg/kg q24h, recommended for CF patients, achieved a 100% PTA in free plasma and 95.8% PTA in free lung, and demonstrated higher PTA in ELF (30.2%) than the 3 mg/kg q24h regimen. None of the treatments evaluated reached PTA ≥ 90% for free ELF concentrations when TOB was intravenously administered.

## 4. Discussion

TOB is used to treat lung infections caused by *P. aeruginosa* in fibrocystic and non-fibrocystic patients. In this work we investigated the impact of biofilm-forming *P. aeruginosa* infection on TOB lung and ELF penetration by developing a preclinical popPK model with data obtained by microdialysis in rodents. The model was used to simulate free lung and ELF concentrations in humans by applying dose allometric scaling. The PTA of the free concentration–time profiles generated by model simulation for different dose regimens to treat *P. aeruginosa* pneumonias was investigated using the PK/PD index ƒC_max_/MIC > 10. To the best of our knowledge, this is the first study that describes pre-clinical free TOB lung and ELF concentrations, determined by microdialysis, in healthy and biofilm-forming *P. aeruginosa*-infected rodents using a population pharmacokinetic approach to investigate the efficacy of TOB dosing regimens to treat pneumonia.

The preclinical popPK model developed demonstrated that the lung alterations in TOB distribution observed in acute and chronic infection caused by biofilm-forming *P. aeruginosa* resulted in significant increase in lung volume (V3) in both infected conditions (V3_infected_ = 0.130 L), compared to the healthy group (V3 = 0.083). As well as increasing lung volume of distribution, chronic infection also increased TOB CL and V1 (Table 1). The group inoculated with blank-bead, used as control, also showed increased lung volume (V3) compared to healthy group, but did not present plasma pharmacokinetic alterations. The animal model of chronic infection used in the present work, produced by *P. aeruginosa* strain (ATCC 27853) embed in alginate beads, is characterized by a lung infection that mimics the chronic pneumonia observed in CF patients, with the presence of mucus [16,17,18,19]. The alterations in TOB lung pharmacokinetics observed in the blank-bead group demonstrate that the alginate present in the lungs of the animals sequester the drug, contributing to the reduced free concentrations observed in the chronically infected group. However, the plasma alterations observed in the chronically infected group were due solely to biofilm-forming *P. aeruginosa* infection. The acute infection, induced by a non-mucoid strain (PA14), also showed an increase in V3. This set of results demonstrates that the infection with biofilm-forming *P. aeruginosa*, independent of the alginate presence at the biophase or the stage of infection progression (acute or chronic), produces alterations in TOB lung disposition.

The increased TOB lung volume (V3) could be attributed to the increased volume of fluids in the lung compartment due to altered capillary permeability and plasma extravasation, in combination with the reduction in amount of drug available due to its binding to the biofilm extracellular matrix components. Tobramycin free concentrations in ELF were proportionally lower than lung concentrations, both in healthy and infected conditions. This enables ELF exposure to TOB to be described as a fraction of the lung compartment exposure. The inclusion of the D_factor_ in the model means that TOB distribution to ELF depends on its free concentration in the lung interstitial fluid, and that any alteration observed at lung level due to the infection will be proportionally reflected in the ELF.

Tobramycin pharmacokinetic alterations in total CL due to *P. aeruginosa* infection in CF patients were already reported by Hong et al. (2018) [28]. They found an increase in TOB CL due to the infection was decreased from day 2 to day 7 after the start of treatment, demonstrating that as long as the patient was recovering from the infection, pharmacokinetic parameters were changing. Furthermore, a popPK model develop by Hennig et al. (2013) [11] using clinical data from CF adult and pediatric patients, as well as from non-CF oncologic patients with febrile neutropenia, did not incorporate CF as a covariate, showing that TOB plasma pharmacokinetics are not impacted by the disease but by the infection itself. The results of the present work corroborate the findings of Hennig et al. (2013) that chronic *P. aeruginosa* infection interferes with TOB plasma distribution and elimination. Because of the alteration of TOB plasma and tissue pharmacokinetics due to infection, strategies for dose adjustment should be considering to warrant better treatment outcomes.

The PTA is used to evaluate the chance that a treatment has to achieve the pharmacological target. The PTA analysis of TOB current dosing regimens using the PK/PD index *f*C_max_/MIC > 10 as target showed that 3 mg/kg q24h produced free lung interstitial concentrations with 85.6% probability of target attainment to treat the most prevalent *P. aeruginosa* MIC (0.5 mg/L), superior to the PTA (62.8%) observed for the same total daily dose fractionated (q8h) (Figure 3). The present result corroborates previous clinical reports that a once-a-day dosing regimen is more effective for treating *P. aeruginosa* lung infection that fractioned dosing and should be preferred for aminoglycosides [12,29]. According to the popPK model estimations, in order to reach a PTA higher than 90% for treating acute infection assuming free lung concentrations, TOB dosing regimen should be 4.5 mg/kg q24h. None of the treatments investigated for acute infection (1 mg/kg q8h and 3 mg/kg q24h) reached sufficient TOB free concentrations in ELF to guarantee a PTA ≥ 90% for the most prevalent *P. aeruginosa* MICs, indicating the need to explore other administration routes.

Hennig et al. (2013) applied a utility function to determine the use of 11 mg/kg 30 min i.v. infusion once daily in CF patients to reach a balance between effective and toxic concentrations. The PTA analysis of this regimen for chronic infection showed a PTA higher than 90% for free plasma (100%) and free lung (95.8%) concentrations at the most prevalent MIC (0.5 mg/L), but did not show sufficient free concentrations in ELF (PTA of 30.2%).

In clinical practice, it is difficult to find a dosing regimen for TOB that combines a high efficacy and low toxicity. Our findings reinforce the superiority of the once-a-day doing regimen for treatment of *P. aeruginosa* lung infection pointing to the need to clinically investigate other doses to increase the PTA to treat acute lung infections. Furthermore, our findings demonstrated that even the 11 mg/kg q24h intravenously administered dose does not produce free ELF concentrations high enough to produce effective treatments. Owing to TOB’s low ELF penetration, other routes of administration must be considered when the objective is bacterial eradication in this biophase.

Despite the fact that the model simulations were performed using doses allometrically scaled to humans, and the free plasma and ELF simulated concentrations were similar to those experimentally determined in patients by Boseli et al. (2007) [24], the lung distribution of TOB in acutely and chronically infected humans still needs to be confirmed by additional clinical studies. Furthermore, the ƒC_max_/MIC target greater than 10 for the PTA analysis of plasma, lung, and ELF concentrations used in this study was previously established for free plasma concentrations [25]. There is no PK/PD index established assuming free TOB tissue concentrations at the biophase, which can be a limitation for the analysis presented here.

The approach used in the present study highlights the importance of assessing antimicrobial free concentrations in infected biophases and combining these data using modeling and simulation to determine adequate doses to reach effective antimicrobial treatments.

## 5. Conclusions

In conclusion, the present study used a popPK approach to describe the alterations observed in TOB plasma, free lung, and free ELF concentrations determined due to acute and chronic biofilm-forming *P. aeruginosa* infection in rodents. The model was able to describe the reduction in TOB free lung concentrations in infected animals and the proportionality between TOB free ELF and free lung concentrations in the healthy and infected conditions. The model simulations were successfully applied to evaluate the PTA of current TOB dosing regimens used in the clinic. The regimens of 4.5 mg/kg q24h for acute and 11 mg/kg q24h for chronic infections reached > 90% PTA in the lung for the most prevalent *P. aeruginosa* MIC (0.5 mg/mL). The model simulations also demonstrated that none of the regimens tested was able to achieve the target PK/PD index ƒC_max_/MIC > 10 in ELF. These results demonstrated the need to investigate alternative administration routes if the goal is to eradicate infection in the ELF.

## Figures and Tables

**Table 1:** Population pharmacokinetic parameters estimate for the popPK model.

Parameter	Estimate (% RSE)	Bootstrap * Median (95% CI)
CL (L/h)	0.047 (12)	0.041 (0.020–0.066)
CL_chronic_ (L/h)	0.085 (23)	0.084 (0.045–0.133)
V1 (L)	0.055 (16)	0.067 (0.044–0.106)
V1c_hronic_ (L)	0.323 (17)	0.424 (0.186–0.470)
Q1 (L/h)	0.030 (8)	0.036 (0.024–0.109)
V2 (L)	0.154 (16)	0.194 (0.083–0.693)
Q2 (L/h)	0.370 (5)	0.366 (0.257–0.475)
V3 (L)	0.083 (29)	0.062 (0.022–0.119)
V3_infected_ (L)	0.130 (22)	0.112 (0.042–0.203)
D_factor_	0.36 (10)	0.36 (0.25–0.50)
ωCL (%CV)	84 (10)	111 (58–313)
ωV1 (%CV)	60 (17)	32 (1–132)
ωV3 (%CV)	134 (11)	218 (120–485)
ωD_factor_ (%CV)	43 (16)	46 (17–83)
Plasma log-additive error (mg/L)	0.152 (5)	0.144 (0.102–0.180)
Microdialysis log-additive error (mg/L)	0.313 (3)	0.43 (0.268–0.595)

CL: total clearance; CL_chronic_: total clearance for chronically infected animals; V1: volume of the central compartment; V1_chronic_: volume of the central compartment for chronically infected animals; Q1, Q2: intercompartmental clearances; V2: volume of the peripheral compartment; V3: volume of the lung compartment for healthy animals; V3_infected_: volume of the lung compartment for acutely and chronically infected and blank-bead animals; D_factor_: distribution factor from lung to ELF. RSE (%): relative standard error; CV: coefficient of variation; CI: confidence interval. Shrinkage values for ωCL: 5.2%; ωV1: 36.5%; ωV3: 12.8%; ωD_factor_: 43.7%. * 960/1000 successful runs.

## Data Availability

The data presented in this study are available upon request to the corresponding author.

## Supplementary Materials

The following supporting information can be downloaded at: https://www.mdpi.com/article/10.3390/pharmaceutics14061237/s1, Figure S1. Graphs from HPLC-MS/MS analysis for microdialysate samples. (A) blank, (B) calibration curve sample for 0.5 μg/mL, and (C) quantified sample. First panel of the sequence is the chromatogram for all analytes, second panel is the chromatogram for tobramycin mass transition, and third panel is the IS mass transition. Figure S2. Graphs from HPLC/MS-MS analysis for plasma samples. (A) blank, (B) calibration curve sample for 0.1 μg/mL, and (C) quantified sample. First panel of the sequence is the chromatogram for all analytes, second panel is the chromatogram for tobramycin mass transition, and third panel is the IS mass transition. Figure S3. Tobramycin concentration–time curves in total plasma, free lung, and free ELF after a 10 mg/kg i.v. bolus dosing to healthy, acutely, and chronically biofilm-forming *P. aeruginosa*-infected groups, and animals inoculated with blank beads (control group). Points are observations. Figure S4. Exploratory analysis of weight (WT) as a continuous covariable on base model for model parameters and interindividual variability with correlation coefficient and *p*-value. Figure S5. Box plot of exploratory analysis of categoric covariables from base model in model parameters and interindividual variability. Table S1. Human dosing regimens allometrically scaled to rat, used in the simulations. Table S2. Number of animals and observations of each experimental group and condition. Figure S6. Goodness-of-fit plots from TOB final popPK showing the observations vs. population and individual predictions, and conditional residuals vs. population predictions and time. Figure S7. Individual pharmacokinetic profiles from observed data (points), and populational and individual predictions (line and dashed line) for the final popPK model for tobramycin total plasma (A), free lung (B), and free ELF (B). Figure S8. Simulated concentration versus time profile for 10 mg/kg dosing of tobramycin, with median (line) and 32nd and 68th percentiles (shadow area) for free plasma and free ELF. Points show free TOB concentration in plasma and ELF of patients extracted from Boselli et al. (2007) [24].

## References

[1] Høiby N., Ciofu O., Johansen H.K., Song Z., Moser C., Jensen P.Ø., Molin S., Givskov M., Tolker-Nielsen T., Bjarnsholt T. (2011). The clinical impact of bacterial biofilms. Int. J. Oral Sci..

[2] Wang H., Wu H., Ciofu O., Song Z., Høibya N. (2012). In Vivo pharmacokinetics/pharmacodynamics of colistin and imipenem in *Pseudomonas aeruginosa* biofilm infection. Antimicrob. Agents Chemother..

[3] Wozniak D.J., Wyckoff T.J.O., Starkey M., Keyser R., Azadi P., O’Toole G.A., Parsek M.R. (2003). Alginate is not a significant component of the extracellular polysaccharide matrix of PA14 and PAO1 *Pseudomonas aeruginosa* biofilms. Proc. Natl. Acad. Sci. USA.

[4] Dhanani J., Roberts J.A., Chew M., Lipman J., Boots R.J., Paterson D.L., Fraser J.F. (2010). Antimicrobial chemotherapy and lung microdialysis: A review. Int. J. Antimicrob. Agents.

[5] Bergongne-Berezin E. (1995). New concepts in the pulmonary disposition of antibiotics. Pumonary Pharmacol..

[6] Bergongne-Berezin E. (1981). Penetration of antibiotics into the respiratory tree. J. Antimicrob. Chemother..

[7] Mazzei T., Novelli A., De Lalla F., Mini E., Periti P. (1995). Tissu penetration and Pulmonary Disposition of Tobramycin. J. Chemother..

[8] Reyes M.P., Zhao J.J., Buensalido J.A.L. (2014). Current Perspectives: Therapeutic Uses of Tobramycin. J. Pharmacovigil..

[9] Lund-Palau H., Turnbull A.R., Bush A., Bardin E., Cameron L., Soren O., Wierre-Gore N., Alton E.W.F.W., Bundy J.G., Connett G. (2016). *Pseudomonas aeruginosa* infection in cystic fibrosis: Pathophysiological mechanisms and therapeutic approaches. Expert Rev. Respir. Med..

[10] Müller L., Murgia X., Siebenbürger L., Börger C., Schwarzkopf K., Sewald K., Häussler S., Braun A., Lehr C.M., Hittinger M. (2018). Human airway mucus alters susceptibility of *Pseudomonas aeruginosa* biofilms to tobramycin, but not colistin. J. Antimicrob. Chemother..

[11] Hennig S., Standing J.F., Staatz C.E., Thomson A.H. (2013). Population pharmacokinetics of tobramycin in patients with and without cystic fibrosis. Clin. Pharmacokinet..

[12] Burkhardt O., Lehmann C., Madabushi R., Kumar V., Derendorf H., Welte T. (2006). Once-daily tobramycin in cystic fibrosis: Better for clinical outcome than thrice-daily tobramycin but more resistance development?. J. Antimicrob. Chemother..

[13] Ciofu O., Tolker-Nielsen T. (2019). Tolerance and resistance of *Pseudomonas aeruginosa* biofilms to antimicrobial agents—How *P. aeruginosa* can escape antibiotics. Front. Microbiol..

[14] Tseng B.S., Zhang W., Harrison J.J., Quach T.P., Song J.L., Penterman J., Singh P.K., Chopp D.L., Packman A.I., Parsek M.R. (2013). The extracellular matrix protects *Pseudomonas aeruginosa* biofilms by limiting the penetration of tobramycin. Environ. Microbiol..

[15] Goodman A.L., Kulasekara B., Rietsch A., Boyd D., Smith R.S., Lory S. (2004). A signaling network reciprocally regulates genes associated with acute infection and chronic persistence in *Pseudomonas aeruginosa*. Dev. Cell.

[16] Growcott E.J., Coulthard A., Amison R., Hardaker E.L., Saxena V., Malt L., Jones P., Grevot A., Poll C., Osborne C. (2011). Characterisation of a refined rat model of respiratory infection with *Pseudomonas aeruginosa* and the effect of ciprofloxacin. J. Cyst. Fibros..

[17] Van Heeckeren A.M., Schluchter M.D. (2002). Murine models of chronic *Pseudomonas aeruginosa* lung infection. Lab. Anim..

[18] Bragonzi A. (2010). Murine models of acute and chronic lung infection with cystic fibrosis pathogens. Int. J. Med. Microbiol..

[19] Yan P., Chen Y., Song Z., Wu H., Kong J., Qin X. (2008). Pathogenic effects of biofilm with chronic *Pseudomonas aeruginosa* lung infection in rats. J. Nanjing Med. Univ..

[20] Torres B.G.S., Helfer V.E., Bernardes P.M., Macedo A.J., Nielsen E.I., Friberg L.E., Dalla Costa T. (2017). Population pharmacokinetic modeling as a tool to characterize the decrease in biofilm lung infection in Wistar rats. Antimicrob. Agents Chemother..

[21] Johansen H.K., Høiby N., Zak O., Sande M.A. (1999). Rat Model of Chronic *Pseudomonas aeruginosa* Lung Infection. Handbook of Animal Models of Infection.

[22] Bernardi P.M., Barreto F., Dalla Costa T. (2017). Application of a LC–MS/MS method for evaluating lung penetration of tobramycin in rats by microdialysis. J. Pharm. Biomed. Anal..

[23] Pachaly J.R. (2006). Terapêutica Por Extrapolação Alométrica. Tratado Animais Selvagens-Med. Veterinária.

[24] Boselli E., Breilh D., Djabarouti S., Guillaume C., Rimmelé T., Gordien J.B., Xuereb F., Saux M.C., Allaouchiche B. (2007). Reliability of mini-bronchoalveolar lavage for the measurement of epithelial lining fluid concentrations of tobramycin in critically ill patients. Intensive Care Med..

[25] Asín-Prieto E., Rodríguez-Gascón A., Isla A. (2015). Applications of the pharmacokinetic/pharmacodynamic (PK/PD) analysis of antimicrobial agents. J. Infect. Chemother..

[26] EUCAST Calibration of Zone Diameter Breakpoints to MIC Values. https://www.eucast.org/ast_of_bacteria/calibration_and_validation/.

[27] Schumock G.T., Raber S.R., Crawford S.Y., Rodvold K.A. (1995). National Survey of Once-Daily Dosing of Aminoglycoside. Pharmacotherapy.

[28] Hong L.T., Liou T.G., Deka R., King J.B., Stevens V., Young D.C. (2018). Pharmacokinetics of Continuous Infusion Beta-lactams in the Treatment of Acute Pulmonary Exacerbations in Adult Patients with Cystic Fibrosis. Chest.

[29] Magréault S., Roy C., Launay M., Sermet-Gaudelus I., Jullien V. (2021). Pharmacokinetic and Pharmacodynamic Optimization of Antibiotic Therapy in Cystic Fibrosis Patients: Current Evidences, Gaps in Knowledge and Future Directions.

