# Supplementary material for: Probability of Target Attainment of Tobramycin Treatment in Acute and Chronic Pseudomonas aeruginosa Lung Infection Based on Preclinical Population Pharmacokinetic Modeling
Source: Pharmaceutics. 2022 Jun 11;14(6):1237. doi: 10.3390/pharmaceutics14061237 (PMC9228144; doi:10.3390/pharmaceutics14061237)
Supplement: Supplementary file 1 [file pharmaceutics-14-01237-s001.zip › pharmaceutics-1734424-supplementary.pdf]

Supplementary Material

Tobramycin quantification

Tobramycin quantification in plasma and microdialysate samples was conducted in a validated LC-MS/MS method previously described (Bernardi, Barreto & Dalla Costa, 2017). The quantification was performed by liquid chromatography (Agilent 1200 system, Agilent, Germany) in tandem with a triple quadrupole mass spectrometry (Sciex, API 5000, ABSciex, Canada). A C_{18} column (Waters X-Terra®, 100 mm × 2.1 mm, particle size 3.5 µm) with C_{18} Phenomenex AJO-428 security guard column was used. The injection volume was 4 µL, and the column was maintained at 40 ± 1 °C. The chromatographic run time was 9.5 min, at a flow rate of 300 µL/min, consisting of gradient of mobile phase A and B, water and acetonitrile, respectively, both containing 10 mM HFBA. The gradient started with 5 % of B, increased to 90% in 4 min, holding for 4 min and returning to the initial condition for 4 min at the end of the analysis. Tobramycin and apramycin (internal standard, IS), were monitored using electrospray ionization in positive mode (ESI+), at mass transitions *m/z* 468.2 > 163.3 for TOB and 540.3 > 217.2 for IS.

For plasma, the bioanalytical curve was prepared by adding 10 µL of TOB working solutions to 90 µL of Wistar rat blank plasma, to obtain the final concentrations of the standard curve: 0.5, 1, 2.5, 5, 10, 25, 50 and 100 µg/mL. 10 µL of the IS standard working solution was added to all plasma samples to obtain a final concentration of 1 µg/mL. The samples were deprotenized by the addition of 100 µL of 15% trichloreacetic acid (TCA) in acetonitrile-water, (50:50, v/v). After centrifugation at 12,000 rpm for 10 min, 10 µL of the supernatant were diluted into 490 µL of HFBA 10 mM, agitated in vortex during 30 s and then 4 µL injected into the chromatographic system.

The bioanalytical curve for microdyalisate was prepared by adding 10 µL of TOB working solutions to 90 µL of Ringer solution, to obtain the final concentrations of the standard curve: 0.01, 0.25, 0.5, 0.75, 1, 2.5, 5, 10, 25 and 50 µg/mL. 10 µL of the IS working solution was added to all microdialysate samples to obtain a final concentration of 1 µg/mL. 10 µL of the samples were diluted into 40 µL of HFBA 10 mM, agitated in vortex during 30 s and then 4 µL injected into the chromatographic system.

The range of quantification was 0.5-50 mg/L for plasma, 0.1-50 mg/L for lung microdialysis and 0.01-50 mg/L for ELF microdialysis. Standard curves were fitted in a linear equation using a weighting factor of 1/x with coefficients of determination ≥ 0.99. Accuracy and precision were within the acceptance criteria (15%) [22].

A)

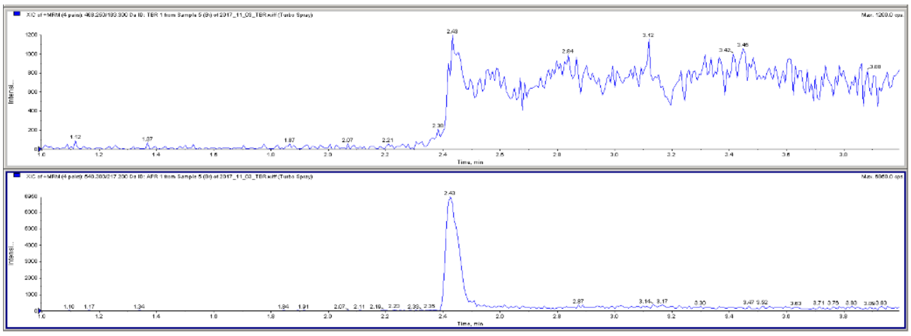

B)

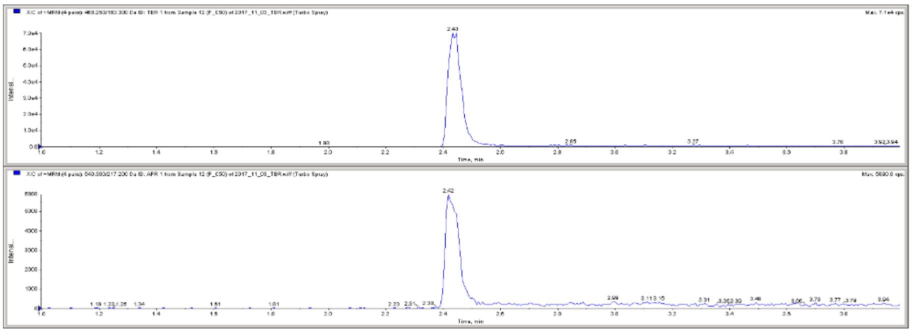

C)

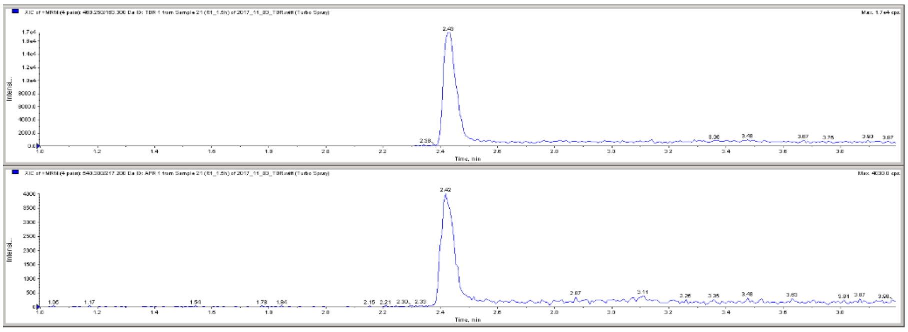

Figure S1. Chromatograms from HPLC/MS-MS analysis for plasma samples: A) blank TOB in upper panel and IS in lower panel, B) calibration curve sample of 0.5 µg/mL of TOB in upper panel and 1 µg/mL of IS in lower panel C) sample collected from rat at 1.5 h after 10 mg/kg TOB i.v. dosing, quantified as 4.4 µg/mL of TOB in upper panel and 1 µg/mL of IS in lower panel.

A)

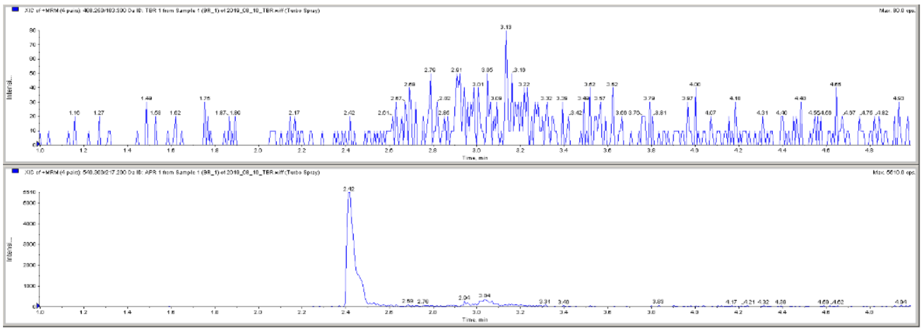

B)

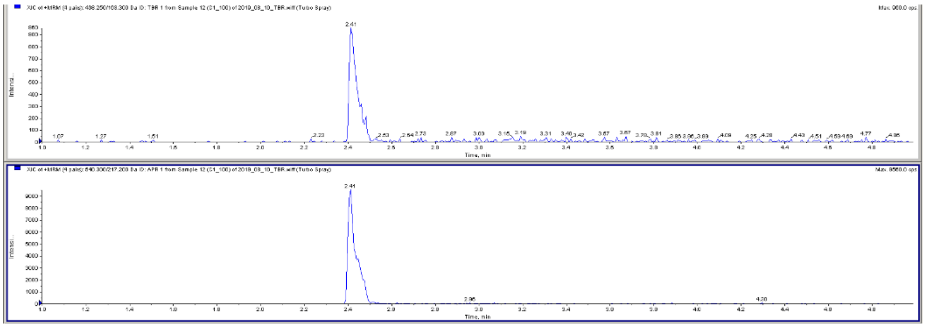

C)

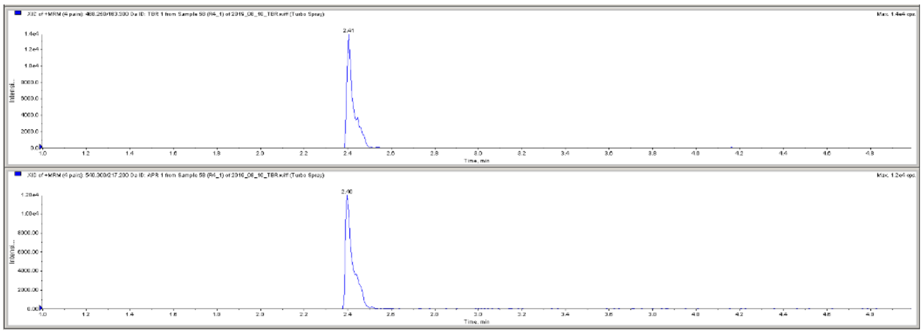

Figure S2. Chromatograms from HPLC/MS-MS analysis for microdialysate samples: A) blank TOB in upper panel and IS in lower panel, B) calibration curve sample of 0.1 µg/mL of TOB in upper panel and 1 µg/mL of IS in lower panel C) sample from rat at first 30 min interval after 10 mg/kg TOB i.v. dosing, quantified as 3.1 µg/mL of TOB in upper panel and 1 µg/mL of IS in lower panel.

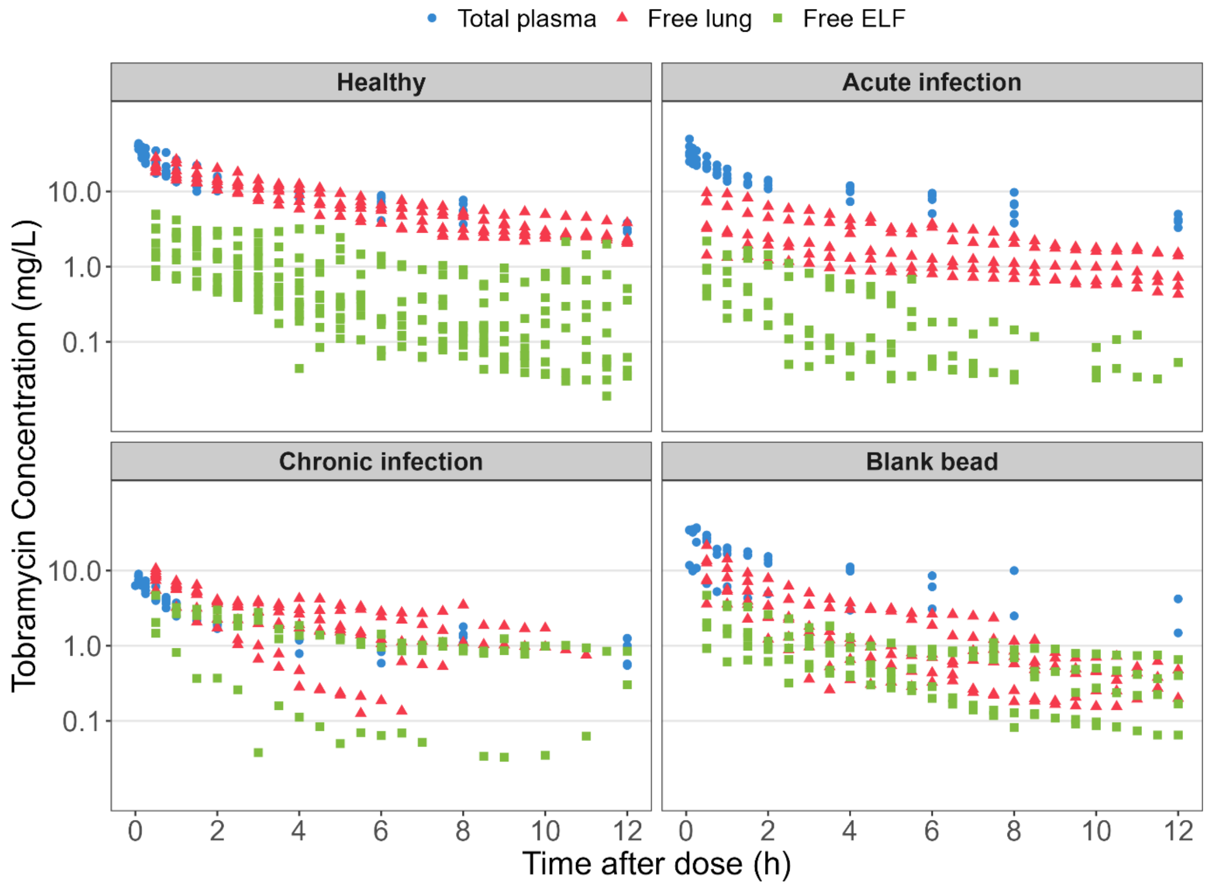

Figure S3. Tobramycin concentration-time curves in total plasma, free lung and free ELF after a 10 mg/kg i.v. *bolus* dosing to healthy, acutely and chronically biofilm-forming *P. aeruginosa* infected groups, and animals inoculated with blank beads (control group). Points are observations.

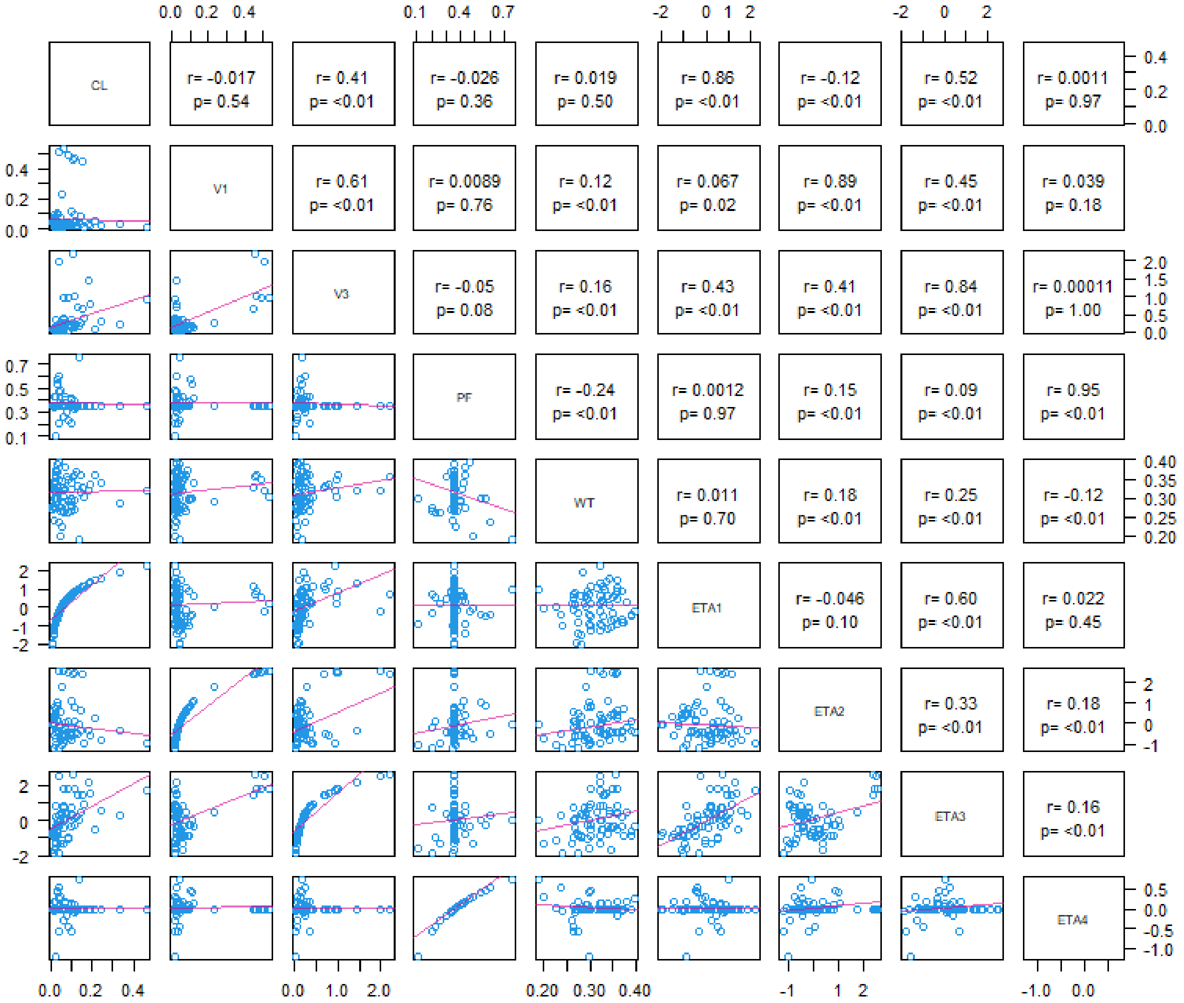

Figure S4. Exploratory analysis of weight (WT) as a continuous covariable on base model for model parameters and interindividual variability with correlation coefficient and p-value.

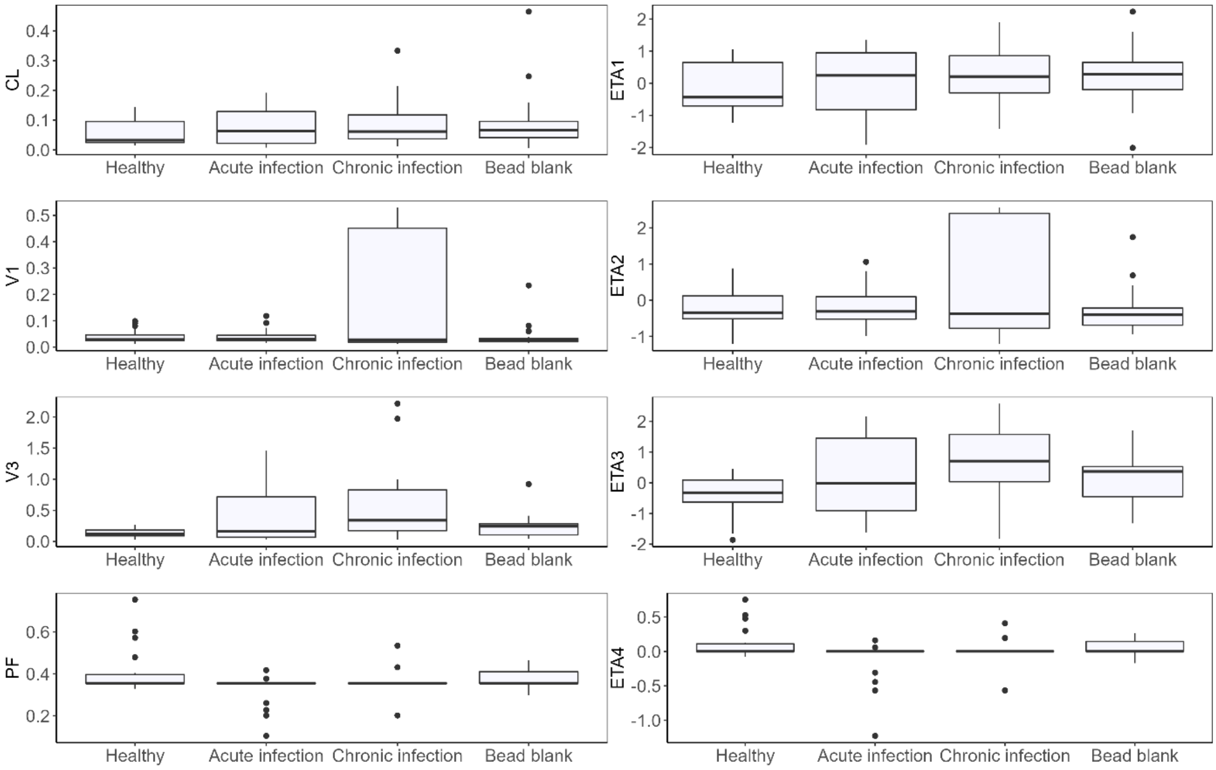

Figure S5. Box plot of exploratory analysis of categoric covariables from base model in model parameters and interindividual variability.

Equations used to perform the allometric scaling:

$$Dose_{rats} = \frac{Dose_{humans}}{BMI_{humans}} \times BMI_{rats} \qquad \text{Eq. S1}$$

$$BMI = K \times M^{0.75} \qquad \text{Eq. S2}$$

where BMI is the body mass index, calculated for humans and rats, with K as the proportionality constant for mean corporal temperature, which for placental mammals assumes a value of 70; M is the body mass, which assumes values of 70 kg for humans and 0.35 kg for rats.

Table S1. Human dosing regimens allometrically scaled to rat, used in the simulations.

Human Dose (mg/kg)	Equivalent Dose in Rats (mg/kg)
1.0	3.8
3.0	11.3
10.0	37.2
11.0	41.4

Table S2. Number of animals and observations of each experimental group and condition.

Groups	Animals/observations
**Plasma**	
Healthy	6/68
Acutely infected	5/59
Chronically infected	6/72
Blank bead blank	4/40
**Lung microdialysis**	
Healthy	6/142
Acutely infected	5/120
Chronically infected	7/106
Blank bead	5/111
**ELF microdialysis**	
Healthy	12/231
Acutely infected	6/91
Chronically infected	3/61
Blank bead	5/111

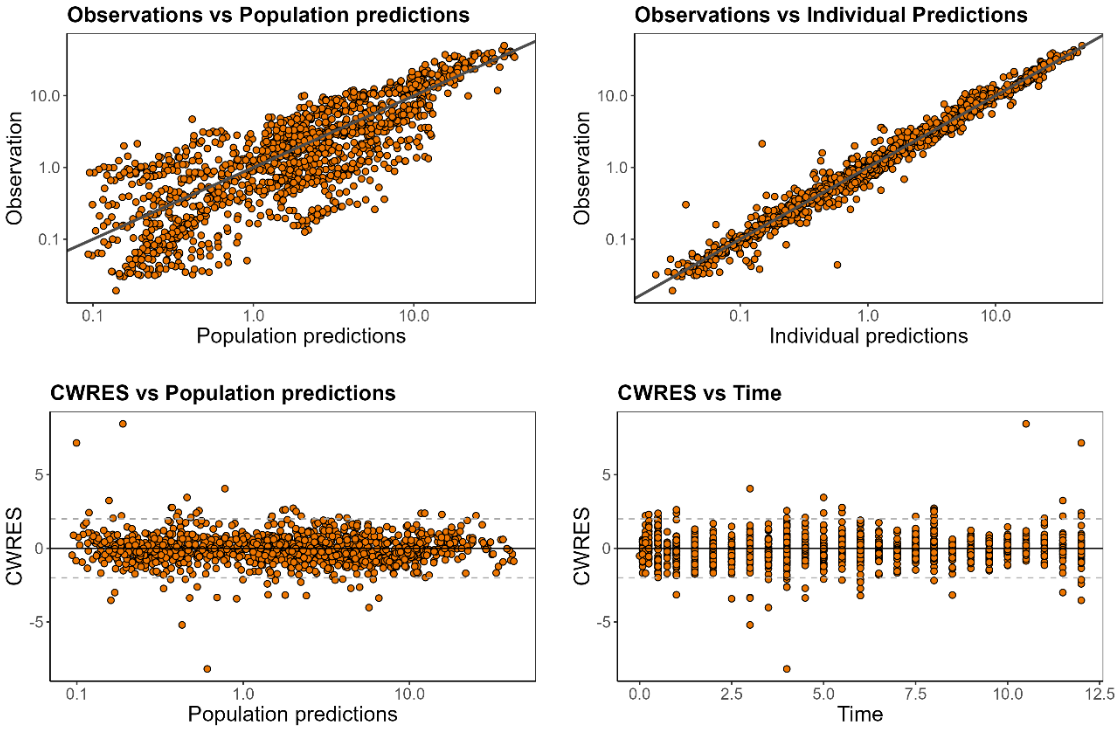

Figure S6. Goodness-of-fit plots from TOB final popPK showing the observations *vs* population and individual predictions, conditional residuals *vs* population predictions and time.

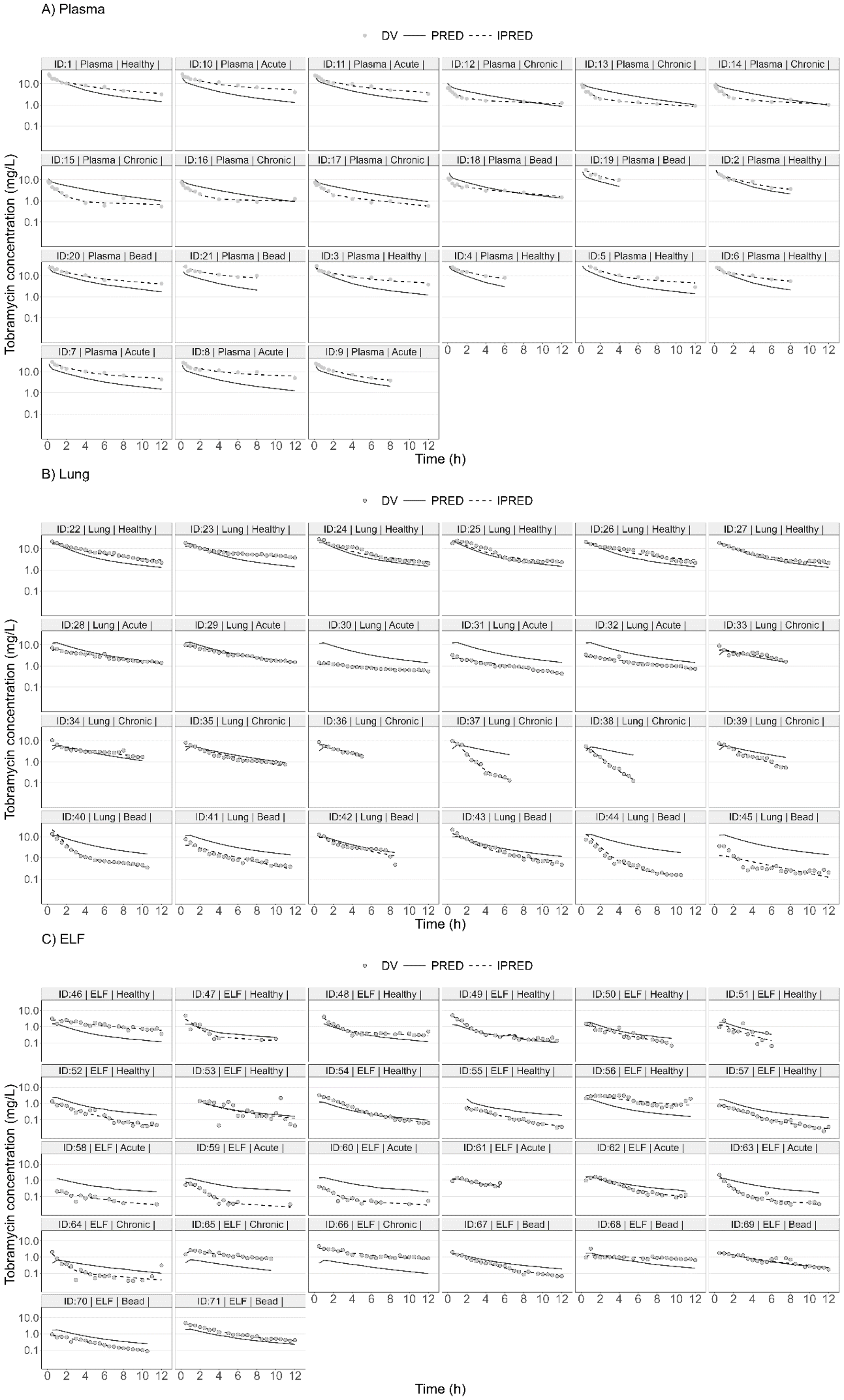

Figure S7. Individual pharmacokinetic profiles from observed data (points), populational and individual predictions (line and dashed line) for the final popPK model for tobramycin total plasma (A), free lung (B) and free ELF (B).

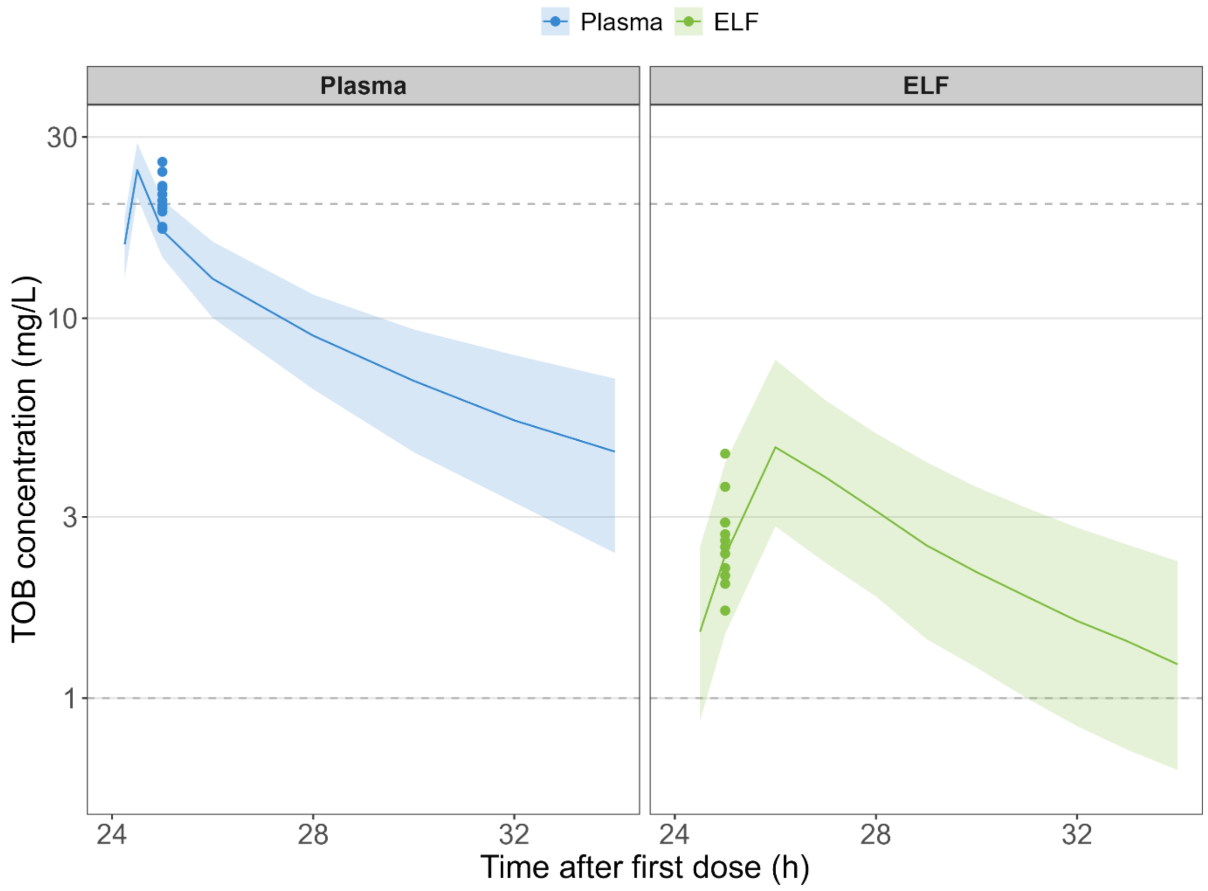

Figure S8. Simulated concentration versus time profile for 10 mg/kg dosing of tobramycin, with median (line) and 32nd and 68th percentiles (shadow area) for free plasma and free ELF. Points show free TOB concentration in plasma and ELF of patients extracted from Boselli et al. (2007).
